# Association between apolipoprotein B/A1 ratio and coronary plaque vulnerability in patients with atherosclerotic cardiovascular disease: an intravascular optical coherence tomography study

**DOI:** 10.1186/s12933-021-01381-9

**Published:** 2021-09-15

**Authors:** Fuxue Deng, Danni Li, Lei Lei, Qiang Yang, Qing Li, Hongtao Wang, Jie Deng, Qiangsun Zheng, Wei Jiang

**Affiliations:** Department of Cardiology, The Second Affiliated Hospital of Xi’an Jiaotong University, Xiwulu 157#, Xi’an, 710004 Shaanxi China

**Keywords:** Optical coherence tomography, Atherosclerotic cardiovascular disease, Apolipoprotein

## Abstract

**Background:**

Apolipoprotein (Apo) A1 and Apo B are strongly associated with the risk of atherosclerotic cardiovascular disease (ASCVD). However, the relationship between the Apo B/A1 ratio and the morphology of coronary vulnerable plaques has not been fully elucidated in patients with ASCVD.

**Methods:**

A total of 320 patients with ASCVD undergoing percutaneous coronary intervention were enrolled and assigned into acute coronary syndrome (ACS) or chronic coronary syndrome (CCS) group. The morphology of culprit plaque was analyzed by intravascular optical coherence tomography. Association between the Apo B/A1 ratio and coronary vulnerable plaques were evaluated using logistic regression models and receiver operator characteristic (ROC) curve analyses.

**Results:**

The Apo B/A1 ratio was higher in ACS patients than CCS patients (0.77 ± 0.28 vs. 0.64 ± 0.22, P < 0.001) and it was also higher in patients with plaque rupture, erosion or thrombus than those without culprit plaques. The high Apo B/A1 ratio was associated with high percent of vulnerable plaques compared with low ratio group. The Apo B/A1 ratio was negatively related to fibrous cap thickness in lipid-rich plaque (r = − 0.228, P = 0.043). Univariate and multivariate logistic regression analyses revealed that the Apo B/A1 ratio was an independent factor of plaque rupture, erosion, and thrombus. The area under the ROC curve of the Apo B/A1 ratio for plaque rupture, erosion, and thrombus were 0.632, 0.624, and 0.670 respectively (P < 0.001 for all), which were higher than that of low-density lipoprotein cholesterol.

**Conclusions:**

The Apo B/A1 ratio is an independent predictor for plaque rupture, erosion, and thrombus in patients with ASCVD.

**Supplementary Information:**

The online version contains supplementary material available at 10.1186/s12933-021-01381-9.

## Background

Apolipoprotein (Apo) A1, a main structural protein of high-density lipoprotein cholesterol (HDL-C), plays a key role in reversing cholesterol flow and cellular cholesterol homeostasis [1]. While Apo B exists as a single protein molecule and serves as an alternative factor to low-density lipoprotein cholesterol (LDL-C) for cardiovascular risk assessment [2]. Recent clinical studies indicate that the Apo B/A1 ratio, which may reflect the cholesterol balance between atherogenic and antiatherogenic lipoprotein particles, is a strong predictor of risk for atherosclerotic cardiovascular disease (ASCVD) [3, 4].

According to the latest guidelines, the dynamic nature of the ASCVD process can be categorized as either acute coronary syndrome (ACS) or chronic coronary syndrome (CCS) [5]. Intra-coronary imaging techniques, including intravascular ultrasound (IVUS), optical coherence tomography (OCT), and fractional flow reserve (FFR), are recommended for percutaneous coronary intervention (PCI) in clinical practice [6]. OCT is a high-resolution intravascular imaging modality that can identify the characteristics of different coronary artery plaques [7]. However, the relationship between Apo B/A1 ratio and the morphology of coronary vulnerable plaques has not been fully elucidated in patients with ASCVD. Therefore, this study aims to investigate the association between them in these settings and to determine whether the Apo B/A1 ratio can serve as a predictor of coronary vulnerable plaques.

## Methods

### Patient population

Patients with ASCVD from the Second Affiliated Hospital of Xi’an Jiaotong University were retrospectively enrolled from August 2019 to December 2020. Data on anthropometry, lifestyle, and medical history were obtained at baseline. Patients (aged ≥ 18) diagnosed with ASCVD and undergoing PCI were screened for OCT examination. The main exclusion criteria were: cardiogenic shock, end-stage renal disease, serious liver dysfunction, allergy to contrast media, and contraindication to aspirin or ticagrelor. Moreover, patients with left main coronary artery disease, chronic total occlusion, extremely tortuous artery, or heavily calcified vessels were excluded because of potential difficulty in performing OCT examination. Left ventricular ejection fraction (LVEF) was evaluated by echocardiography using modified Simpson’s method within 24 h upon admission.

According to ASCVD guidelines, ACS includes ST-segment elevation myocardial infarction (STEMI), non-ST-segment elevation myocardial infarction (NSTEMI), and unstable angina (UA). CCS are those different evolutionary phases of ASCVD, such as stable angina, asymptomatic and symptomatic patients > 1 year after initial diagnosis or revascularization, suspected vasospastic or microvascular disease, but excluding the situations of ACS [5].

### Blood collection

Blood samples were taken into tubes containing 0.1% EDTA for serum lipid analyses at the first time when patients were hospitalized. The concentrations of Apo and other relevant lipoprotein markers, such as LDL-C, HDL-C, total cholesterol (TC), and total triglycerides (TG), were measured by electro-chemiluminescence immunoassay (Roche Diagnostics, Indianapolis, IN). Other laboratorial parameters were measured according to standard test protocols.

### Angiographic procedure

Coronary angiography was performed via the transradial or transfemoral approach with a 6F or 7F sheath. Intravascular infusion of 70–100 IU/kg unfractionated heparin was given prior to PCI. The culprit vessel was determined by coronary angiography results and electrocardiogram or echocardiographic information with proof of possible myocardial ischemia.

### OCT imaging and analysis

A commercially available frequency-domain OCT system (OCT Mobile Dragonfly, St. Jude Medical/Abbott, St. Paul, MN, USA) was used in the study. After intracoronary administration of 0.2 mg nitroglycerin, an OCT imaging catheter was advanced distal to the lesion and automated pullback was initiated at a rate of 20 mm/s after flushing with contrast media manually from the guiding catheter to create a virtually blood-free environment. The total length of OCT pullback was 75 mm. Thrombus aspiration and/or gentle pre-dilation with small balloon were applied for acute total occluded lesions or severe stenosis lesions as per need to make sure that the OCT catheter could pass through smoothly.

Definitions of image characteristics on OCT were based mainly on previous consensus [7]. Culprit plaques were classified as fibrous plaque with homogeneous and highly backscattering regions (Fig. 2a) or lipid-rich plaque with low signal regions and diffuse borders (Fig. 2b). The fibrous cap thickness (FCT) of a fibrous plaque or lipid-rich plaque was measured three times and the average value was calculated. The lipid arc was measured at 1-mm intervals throughout the entire lesion, and the largest arc was recorded. Calcification within plaques was identified by the presence of sharply delineated, low-backscattering heterogeneous regions (Fig. 2c). Calcium arc and depth were measured using cross-sectional OCT images at 1-mm intervals. The length of the calcification lesion was measured as the span of the entire culprit plaque in the longitudinal view. Thin-cap fibroatheroma (TCFA) was defined as a lipid-rich plaque with the thinnest part of the fibrous cap being less than 65 μm (Fig. 2d).

**Fig. 1:**
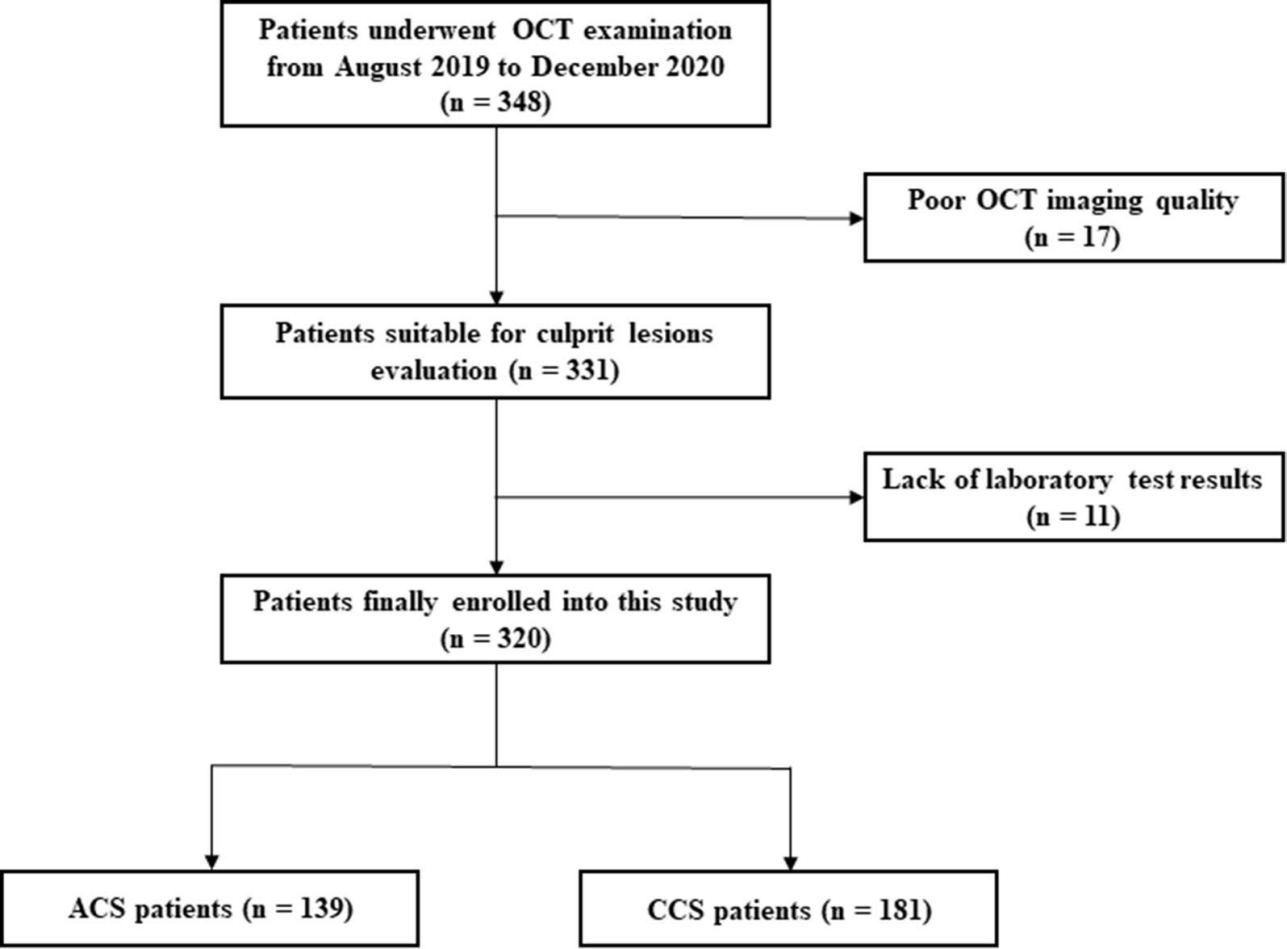
Study flow chart of this study. OCT, optical coherence tomography; hs-cTnI, high-sensitivity cardiac troponin I; *ACS* acute coronary syndrome, *CCS* chronic coronary syndrome

**Fig. 2:**
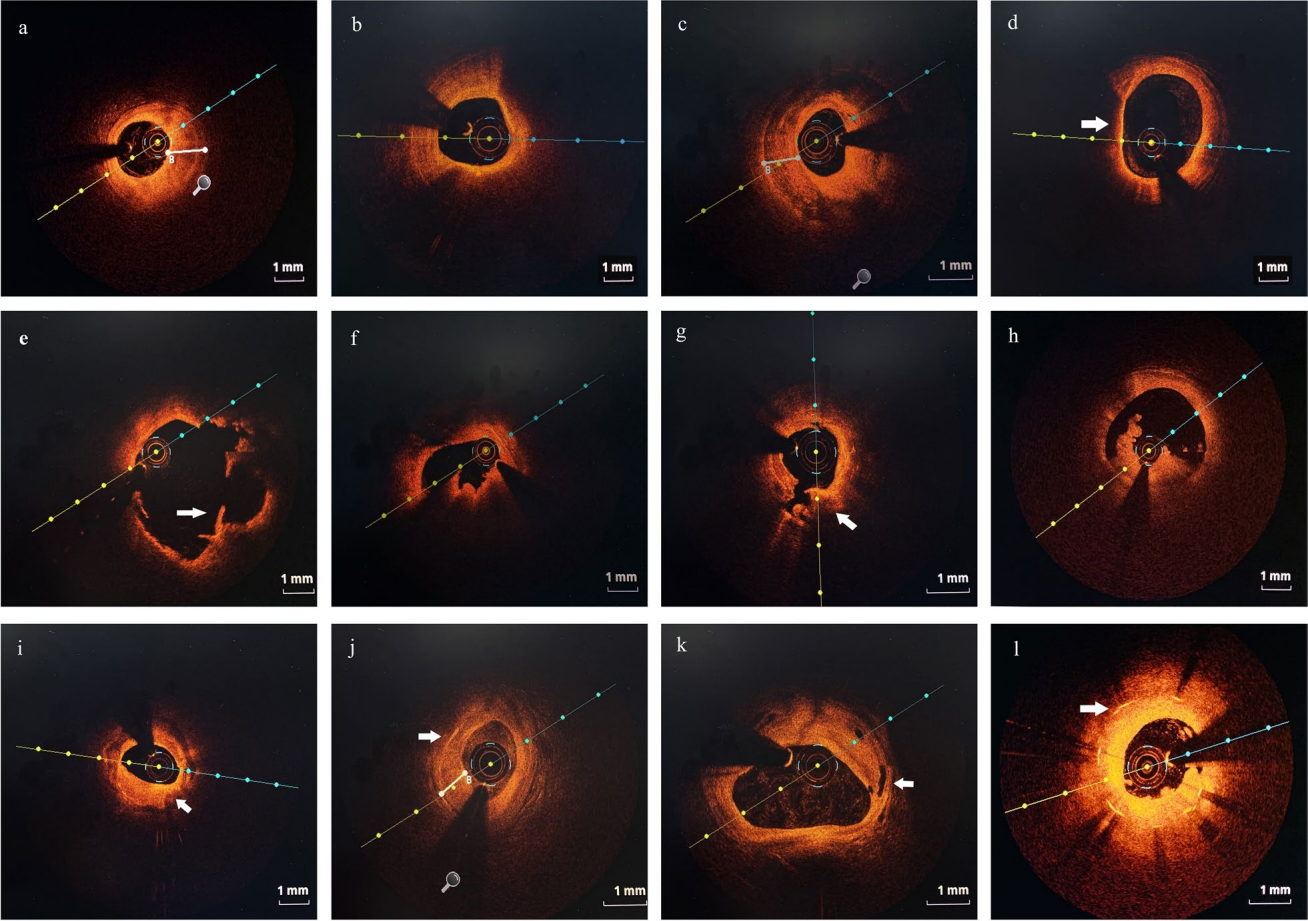
Representative cross-sectional optical coherence tomography images of culprit vessels. **a** Fibrous plaque identified as homogeneous and highly backscattering region. **b** Lipid-rich plaque identified as a low signal region with a diffuse border. **c** Calcification identified as sharply-delineated, low-backscattering heterogeneous regions. **d** Thin-cap fibroatheroma (TCFA) identified as a lipid-rich plaque with the thinnest part of the fibrous cap being less than 65 μm (arrow). **e** Plaque rupture identified as disruption of the fibrous cap with obvious cavity formation (arrow). **f** Plaque erosion identified as the presence of attached thrombus overlying an intact and visualized plaque. **g** Calcified nodule identified as a nodular calcification that protruded to the lumen with thrombus formation (arrow). **h** Thrombus defined as irregular mass that adhered to the luminal surface including white thrombus, red thrombus, and mixed thrombus. **i** Macrophages defined as signal-rich, distinct or confluent punctuate regions with heterogeneous backward shadows (arrow). **j** Cholesterol crystals defined as linear, highly backscattering structures within the plaque (arrow). **k** Microvessels defined as black holes within a plaque that were presented on at least three consecutive frames (arrow). **l** In-stent restenosis was defined as stenosis greater than 50% of the vessel lumen diameter (arrow)

Plaque rupture was identified by disruption of the fibrous cap with obvious cavity formation (Fig. 1e). Plaque erosion was defined as the presence of attached thrombus overlying an intact and visualized plaque (Fig. 1f). Calcified nodule was recognized as a nodular calcification that protruded to the lumen with thrombus formation (Fig. 2g). Thrombus was defined as irregular mass that adhered to the luminal surface including white thrombus, red thrombus, and mixed thrombus (Fig. 2h).

Macrophages were defined as signal-rich, distinct or confluent punctuate regions with heterogeneous backward shadows (Fig. 2i). Cholesterol crystals were defined as linear, highly backscattering structures within the plaque (Fig. 2j). Microvessels were defined as black holes within a plaque that were presented on at least three consecutive frames (Fig. 2k). In-stent restenosis was defined as stenosis greater than 50% of the vessel lumen diameter after stent was implanted (Fig. 2l). All OCT images were analyzed on an OCT workstation by three independent investigators who were blinded to angiographic and clinical data.

### Statistical analysis

All analyses were performed using SPSS 26.0 statistical software (SPSS Inc, Chicago, IL, USA). Continuous data are presented as mean ± standard deviation (SD) and were compared using independent samples *t*-test or Mann–Whitney *U* test between two groups. Categorical data are presented as number (%), and were compared using the Chi-square or Fisher’s exact test. Correlations between variables were determined using the Pearson test or Spearman’s rank test, as appropriate. Logistic regression analyses with adjustments for confounding factors were used to determine association of Apo B/A1 ratio with vulnerable-plaques. The predictive value of the Apo B/A1 ratio for plaque rupture, erosion, and thrombus was further examined by receiver operator characteristic (ROC) curve analysis. A two-tailed P value less than 0.05 was considered statistically significant.

## Results

### Baseline characteristics

We enrolled 348 patients who presented with ASCVD and underwent OCT examination from August 2019 to December 2020 in our hospital. Among these patients, 28 patients were excluded because of poor OCT imaging quality (n = 17) and lack of enough laboratory test results (n = 11). Finally, 320 patients were enrolled into this study and classified into the ACS (n = 139) and CCS (n = 181) groups according to clinical diagnosis and their clinical presentation. The study flow chart is displayed in Fig. 1.

Of the 320 enrolled patients, 77.8% were males, and the mean age was 59.7 years. The comparison of baseline clinical characteristics between ACS and CCS groups is listed in Table 1. The angiographic findings of culprit vessels and number of stents implanted into these patients were also presented. No significant differences were observed in terms of age, medical history or alcohol drinking between ACS and CCS groups. However, more males, higher percentage of smoking habits, and longer smoking years were presented in ACS group. The acute phase biomarkers including white blood cells (WBCs) and myocardial injury markers were also higher in ACS group, which were consistent with the pathogenesis of ACS. In terms of serum lipid profile, the concentrations of TC, HDL-C, LDL-C, lipoprotein (a), Apo A1, Apo B, and the Apo B/A1 ratio were significantly higher in ACS group than that in the CCS group (P < 0.05). However, the LVEF was lower in ACS group, indicating the reduced systolic function of the heart in patients with ACS (P < 0.001). No difference was found in the statins taken at admission between the two groups (P = 0.531).

**Table 1:** Baseline clinical and angiographic characteristics of patients in ACS and CCS groups

Characteristics	All (*n* = 320)	ACS (*n* = 139)	CCS (*n* = 181)	P value
Male, n (%)	249 (77.8%)	121 (87.1%)	128 (70.7%)	< 0.001
Age, years, mean ± SD	59.70 ± 10.45	58.99 ± 11.88	60.25 ± 9.21	0.283
Medical history, n (%)				
Atrial fibrillation	11 (3.4%)	10 (7.2%)	1 (0.6%)	0.001
Hypertension	166 (51.9%)	79 (56.8%)	87 (48.1%)	0.120
Diabetes mellitus	80 (25.0%)	32 (23.0%)	48 (26.5%)	0.474
ASCVD	148 (46.3%)	60 (43.2%)	88 (48.6%)	0.332
Prior PCI	65 (20.3%)	30 (21.6%)	35 (19.3%)	0.621
Stroke	19 (5.9%)	10 (7.2%)	9 (5.0%)	0.404
Alcohol drinking	50 (15.6%)	28 (20.1%)	22 (12.2%)	0.051
Smoking	141 (44.1%)	71 (51.1%)	70 (38.7%)	0.027
Smoking years	12.90 ± 15.94	15.14 ± 16.36	11.17 ± 15.43	0.027
Laboratory results, mean ± SD				
WBC, × 10^9^/L	7.00 ± 2.36	7.89 ± 2.84	6.32 ± 1.63	< 0.001
Hb, g/L	140.76 ± 17.51	138.63 ± 19.88	142.39 ± 15.30	0.057
HbA1c, %	6.48 ± 1.70	6.43 ± 1.76	6.52 ± 1.65	0.671
NT-proBNP, pg/ml	670.25 ± 1906.92	1345.28 ± 2727.79	147.76 ± 296.54	< 0.001
Hs-cTnI, pg/ml	292.11 ± 978.31	631.49 ± 1376.67	7.93 ± 2.82	< 0.001
CK-MB, U/L	25.74 ± 35.65	37.86 ± 44.50	16.40 ± 23.04	< 0.001
Glucose, mmol/L	6.15 ± 2.40	6.39 ± 2.91	5.98 ± 1.99	0.266
Creatine, μmol/L	77.10 ± 80.41	91.36 ± 119.68	66.15 ± 14.16	0.005
eGFR, ml/min/1.73m^2^	104.43 ± 28.09	102.07 ± 33.01	106.24 ± 23.57	0.188
Uric acid, μmol/L	312.55 ± 80.69	332.93 ± 83.66	297.46 ± 75.18	< 0.001
TC, mmol/L	3.79 ± 0.99	3.95 ± 1.01	3.68 ± 0.95	0.016
TG, mmol/L	1.71 ± 1.35	1.85 ± 1.55	1.61 ± 1.17	0.116
HDL-C, mmol/L	1.06 ± 0.26	1.02 ± 0.24	1.08 ± 0.26	0.023
LDL-C, mmol/L	2.23 ± 0.84	2.42 ± 0.87	2.09 ± 0.80	< 0.001
VLDL-C, mmol/L	0.52 ± 0.47	0.53 ± 0.51	0.51 ± 0.45	0.663
Apo A1, g/L	1.25 ± 0.26	1.20 ± 0.23	1.29 ± 0.27	0.001
Apo B, g/L	0.84 ± 0.25	0.89 ± 0.27	0.81 ± 0.23	0.003
Apo B/A1	0.70 ± 0.25	0.77 ± 0.28	0.64 ± 0.22	< 0.001
Lipoprotein (a), mg/dl	23.24 ± 28.42	25.05 ± 28.99	21.84 ± 27.98	0.319
TSH, μIU/ml	3.51 ± 5.12	3.25 ± 5.09	3.71 ± 5.15	0.439
D-dimer, μg/ml	564.49 ± 1354.78	749.41 ± 1765.73	422.49 ± 903.22	0.032
LVEF, %	62.82 ± 7.81	60.77 ± 7.76	64.50 ± 7.46	< 0.001
Culprit vessels, n (%)				
LAD	191 (59.7%)	70 (50.4%)	121 (66.9%)	
LCX	40 (12.5%)	20 (14.4%)	20 (11.0%)	
RCA	56 (17.5%)	32 (23.0%)	24 (13.3%)	
Lesion site, n (%)				
Proximal	165 (51.6%)	72 (51.8%)	93 (51.4%)	
Middle	109 (34.1%)	41 (29.5%)	68 (37.6%)	
Distal	13 (4.1%)	9 (6.5%)	4 (2.2%)	
Stents, n (%)				
0	95 (29.7%)	28 (20.1%)	67 (37.0%)	
1	124 (38.8%)	55 (39.6%)	69 (38.1%)	
2	67 (20.9%)	30 (21.6%)	37 (20.4%)	
3	21 (6.6%)	16 (11.5%)	5 (2.8%)	
> 3	13 (4.1%)	10 (7.2%)	3 (1.7%)	
Statins, n (%)	208 (65.0%)	93 (66.9%)	115 (63.5%)	0.531

*ACS* acute coronary syndrome, *CCS* chronic coronary syndrome, *ASCVD* atherosclerotic cardiovascular disease, *PCI* percutaneous coronary intervention, *SD* standard deviation, *WBC* white blood cell, *Hb* hemoglobin, *HbA1c* glycosylated hemoglobin, *NT-proBNP* N-terminal B-type natriuretic peptide, *Hs-cTnI* high-sensitivity cardiac troponin I, *CK-MB* creatine kinase-MB, *eGFR* estimated glomerular filtration rate, *TC* total cholesterol, *TG* total triglycerides, *HDL-C* high-density lipoprotein cholesterol, *LDL-C* low-density lipoprotein cholesterol, *VLDL-C* very low-density lipoprotein cholesterol, *Apo* apolipoprotein, *TSH* thyroid stimulating hormone, *LVEF* left ventricular ejection fraction, *LAD* left anterior descending artery, *LCX* left circumflex artery, *RCA* right coronary artery

### OCT findings of culprit plaques

The detailed comparisons of OCT findings between ACS and CCS patients are listed in Table 2. The percentage of plaque rupture was higher than erosion and calcified nodules both in ACS and CCS groups, while the percentage of vulnerable plaques, including rupture, erosion, and thrombus, was higher in ACS group than that in the CCS group (P < 0.001) except for calcified nodule. No significant difference was observed between them in other common plaque types, such as fibrous plaque, lipid-rich plaque, and calcification plaques. Furthermore, the ratio of Apo B/A1 was higher in plaque rupture, erosion, and thrombus groups than that in non-vulnerable plaque groups (P < 0.001 or P < 0.05) (Table 3).

**Table 2:** Optical coherence tomography characteristics of patients in ACS and CCS groups

Characteristics	All (*n* = 320)	ACS (*n* = 139)	CCS (*n* = 181)	P value
Plaque morphology, n (%)				
Plaque rupture	79 (24.7%)	50 (36.0%)	29 (16.0%)	< 0.001
Plaque erosion	43 (13.4%)	31 (22.3%)	12 (6.6%)	< 0.001
Calcified nodule	12 (3.8%)	6 (4.3%)	6 (3.3%)	0.640
Plaque type, n (%)				
Thrombus	89 (27.8%)	60 (43.2%)	29 (16.0%)	< 0.001
Red thrombus	7 (2.2%)	4 (2.9%)	3 (1.7%)	
White thrombus	53 (16.6%)	34 (24.5%)	19 (10.5%)	
Mixed thrombus	29 (9.1%)	22 (15.8%)	7 (3.9%)	
TCFA	16 (5.0%)	7 (5.0%)	9 (5.0%)	0.979
Fibrous plaque	137 (42.8%)	58 (41.7%)	79 (43.6%)	0.731
FCT of fibrous plaque, μm	960.99 ± 323.80	954.34 ± 300.78	965.57 ± 340.60	0.847
Calcification	86 (26.9%)	33 (23.7%)	53 (29.3%)	0.268
Angle, °	183.46 ± 115.16	198.42 ± 112.44	173.79 ± 117.04	0.357
Thickness, mm	0.84 ± 0.29	0.79 ± 0.33	0.88 ± 0.26	0.218
Length, mm	22.99 ± 12.10	22.83 ± 10.06	23.09 ± 13.36	0.931
Lipid-rich plaque	79 (24.7%)	28 (20.1%)	51 (28.2%)	0.099
FCT, μm	147.39 ± 125.66	145.97 ± 141.54	148.24 ± 116.62	0.938
Lipid arc, °	188.86 ± 66.37	202.62 ± 59.50	180.88 ± 69.36	0.162
Cholesterol crystal	137 (42.8%)	63 (45.3%)	74 (40.9%)	0.426
Micro-vessel	63 (21.6%)	24 (17.3%)	45 (24.9%)	0.101
Macrophage	61 (19.1%)	27 (19.4%)	34 (18.8%)	0.885
In-stent restenosis, n (%)	43 (13.4%)	21 (15.1%)	22 (12.2%)	0.443
Quantitative of target vessel				
MLA, mm^2^	2.24 ± 1.52	2.16 ± 1.61	2.30 ± 1.45	0.440
MLD, mm	1.61 ± 0.48	1.56 ± 0.51	1.64 ± 0.46	0.218
Diameter stenosis, %	43.16 ± 12.99	44.91 ± 13.04	41.86 ± 12.84	0.050
Area stenosis, %	66.56 ± 16.31	68.59 ± 15.92	65.04 ± 16.49	0.070
Reference vessel diameter, mm	2.53 ± 0.66	2.48 ± 0.72	2.56 ± 0.61	0.335
Post-stent MLA, mm^2^	4.87 ± 2.01	4.89 ± 1.75	4.85 ± 2.23	0.914

*ACS* acute coronary syndrome, *CCS* chronic coronary syndrome, *TCFA* thin-cap fibroatheroma, *FCT* fibrous cap thickness, *MLA* minimal lumen area, *MLD* minimal lumen diameter

**Table 3:** Apo B/A1 ratio and OCT vulnerable plaque characteristics in patients

Characteristics	Apo B/A1		P value
Plaque rupture			
Yes	78	0.78 ± 0.28	< 0.001
No	239	0.67 ± 0.24	
Plaque erosion			
Yes	43	0.78 ± 0.25	0.027
No	274	0.69 ± 0.25	
Calcified nodule			
Yes	12	0.66 ± 0.19	0.606
No	305	0.70 ± 0.26	
TCFA			
Yes	16	0.69 ± 0.15	0.833
No	301	0.70 ± 0.26	
Fibrous plaque			
Yes	131	0.71 ± 0.24	0.423
No	181	0.69 ± 0.26	
Calcification			
Yes	85	0.63 ± 0.22	0.002
No	232	0.72 ± 0.26	
Lipid-rich plaque			
Yes	78	0.70 ± 0.25	0.906
No	239	0.70 ± 0.26	
Cholesterol crystal			
Yes	136	0.72 ± 0.25	0.184
No	181	0.68 ± 0.26	
Micro-vessel			
Yes	66	0.72 ± 0.28	0.370
No	251	0.69 ± 0.25	
Thrombus			
Yes	88	0.79 ± 0.28	< 0.001
No	229	0.66 ± 0.23	
Macrophage			
Yes	60	0.70 ± 0.26	0.991
No	257	0.70 ± 0.25	
In-stent restenosis			
Yes	43	0.67 ± 0.22	0.495
No	274	0.70 ± 0.26	

*Apo* apolipoprotein, *TCFA* thin-cap fibroatheroma

Furthermore, we divided subjects into two groups according to the median value of the Apo B/A-1 ratio: low ratio (< 0.67, n = 160, 50.0%) and high ratio (≥ 0.67, n = 160, 50.0%). Detailed baseline characteristics, such as clinical, laboratorial and OCT morphological information were compared between two groups (Table 4). Patients with high Apo B/A1 ratio were younger males with fewer diabetes mellitus (DM). The high ratio group was associated with high percent of plaque rupture, erosion, and thrombus, but not calcified nodule. No difference was found in the statins taken between the two groups (P = 0.160).

**Table 4:** Baseline characteristic of patients in the low and high Apo B/A1 ratio groups

Characteristics	Low ratio (*n* = 160)	High ratio (*n* = 160)	P value
Male, n (%)	110 (68.8%)	139 (86.9%)	< 0.001
Age, years, mean ± SD	61.87 ± 10.10	57.54 ± 10.38	< 0.001
Medical history, n (%)			
Atrial fibrillation	7 (4.4%)	4 (2.5%)	0.357
Hypertension	91 (56.9%)	75 (46.9%)	0.073
Diabetes mellitus	51 (31.9%)	29 (18.1%)	0.005
ASCVD	78 (48.8%)	70 (43.8%)	0.370
Prior PCI	33 (20.6%)	32 (20.0%)	0.889
Stroke	10 (6.3%)	9 (5.6%)	0.813
Laboratory results, mean ± SD			
HbA1c, %	6.42 ± 1.59	6.54 ± 1.80	0.530
NT-proBNP, pg/ml	607.48 ± 1671.90	734.63 ± 2124.78	0.556
Hs-cTnI, pg/ml	249.97 ± 1060.65	333.98 ± 890.54	0.454
TC, mmol/L	3.28 ± 0.76	4.30 ± 0.92	< 0.001
TG, mmol/L	1.37 ± 0.68	2.04 ± 1.71	< 0.001
HDL-C, mmol/L	1.15 ± 0.27	0.96 ± 0.20	< 0.001
LDL-C, mmol/L	1.72 ± 0.62	2.73 ± 0.72	< 0.001
VLDL-C, mmol/L	0.42 ± 0.26	0.62 ± 0.60	< 0.001
Lipoprotein (a), mg/dl	19.56 ± 24.56	26.85 ± 31.42	0.022
LVEF, %	63.12 ± 7.17	62.52 ± 8.40	0.529
Plaque morphology, n (%)			
Plaque rupture	27 (16.9)	52 (32.5%)	0.001
Plaque erosion	12 (7.5%)	31 (19.4%)	0.002
Calcified nodule	7 (4.4%)	5 (3.1%)	0.556
Thrombus	30 (18.8%)	59 (36.9%)	< 0.001
TCFA	6 (3.8%)	10 (6.3%)	0.305
Fibrous plaque	64 (40.0%)	73 (45.6%)	0.309
FCT, μm	955.02 ± 347.42	966.28 ± 303.87	0.844
Calcification	56 (35.0%)	30 (18.8%)	0.001
Angle, °	171.09 ± 122.51	208.65 ± 95.80	0.142
Thickness, mm	0.82 ± 0.27	0.89 ± 0.33	0.310
Length, mm	22.30 ± 11.18	24.48 ± 14.02	0.489
Lipid-rich plaque	39 (24.4%)	40 (25.0%)	0.897
FCT, μm	181.46 ± 154.11	114.98 ± 80.11	0.020
Lipid arc, °	191.38 ± 72.15	186.40 ± 61.03	0.741
Cholesterol crystal	64 (40.0%)	73 (45.6%)	0.309
Micro-vessel	32 (20.0%)	37 (23.1%)	0.497
Macrophage	34 (21.3%)	27 (16.9%)	0.319
Stents, n (%)			
0	56 (35.0%)	39 (24.4%)	
1	57 (35.6%)	67 (41.9%)	
2	29 (18.1%)	38 (23.8%)	
3	11 (6.9%)	10 (6.3%)	
> 3	7 (4.4%)	6 (3.8%)	
Statins, n (%)	98 (61.3%)	110 (68.8%)	0.160

*ASCVD* atherosclerotic cardiovascular disease, *PCI* percutaneous coronary intervention, *SD* standard deviation, *HbA1c* glycosylated hemoglobin, *NT-proBNP* N-terminal B-type natriuretic peptide, *Hs-cTnI* high-sensitivity cardiac troponin I, *TC* total cholesterol, *TG* total triglycerides, *HDL-C* high-density lipoprotein cholesterol, *LDL-C* low-density lipoprotein cholesterol, *VLDL-C* very low-density lipoprotein cholesterol, *Apo* apolipoprotein, *LVEF* left ventricular ejection fraction, *TCFA* thin-cap fibroatheroma, *FCT* fibrous cap thickness

**Fig. 3:**
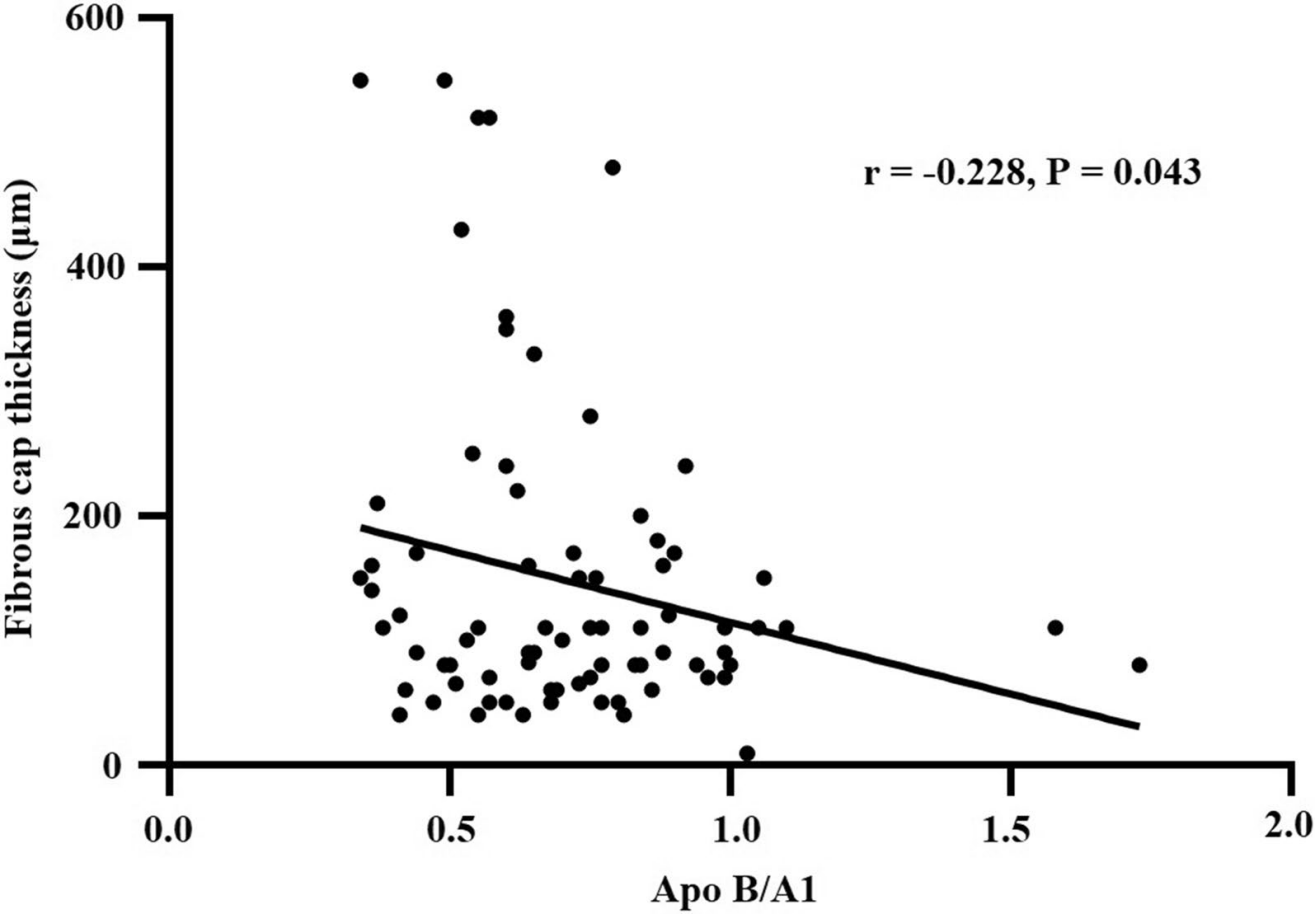
Correlation between Apo B/A1 ratio and fibrous cap thickness in lipid-rich plaque

The correlation study showed that the Apo B/A1 ratio was negatively related to the FCT in lipid-rich plaque (r = − 0.228, P = 0.043) (Fig. 3). Univariate logistic regression analysis revealed that the Apo B/A1 ratio was closely associated with plaque rupture (Table 5), erosion (Additional file 1: Table S1), and thrombus (Additional file 2: Table S2). After adjusting for confounding factors, such as age, sex, medical history, alcohol drinking, and smoking in different models for multivariate logistic regression analysis, the Apo B/A1 ratio remained predictive for plaque rupture, erosion, and thrombus (P < 0.05).

**Table 5:** Logistic regression analysis of plaque rupture

Variables	OR	95% CI	P value
Apo B/A1	5.499	2.019–14.972	0.001
Apo B	4.776	1.716–13.288	0.003
Apo A1	0.470	0.163–1.359	0.163
TG	1.151	0.968–1.368	0.110
TC	1.412	1.094–1.821	0.008
HDL-C	0.473	0.167–1.345	0.160
LDL-C	1.537	1.137–2.078	0.005
VLDL-C	1.524	0.921–2.523	0.101
Lipoprotein (a)	1.008	1.000–1.016	0.064
Model 1	6.257	2.232–17.541	< 0.001
Model 2	4.547	1.583–13.067	0.005
Model 3	4.927	1.676–14.481	0.004

Model 1: Apo B/A1, AF, HBP and DM

Model 2: Apo B/A1, AF, HBP, DM, age and sex

Model 3: Apo B/A1, AF, HBP, DM, age, sex, alcohol drinking and smoking

*Apo* apolipoprotein, *TG* total triglycerides, *TC* total cholesterol, *HDL-C* high-density lipoprotein cholesterol, *LDL-C* low-density lipoprotein cholesterol, *VLDL-C* very low-density lipoprotein cholesterol, *OR* odds ratio, *CI* confidence interval

**Fig. 4:**
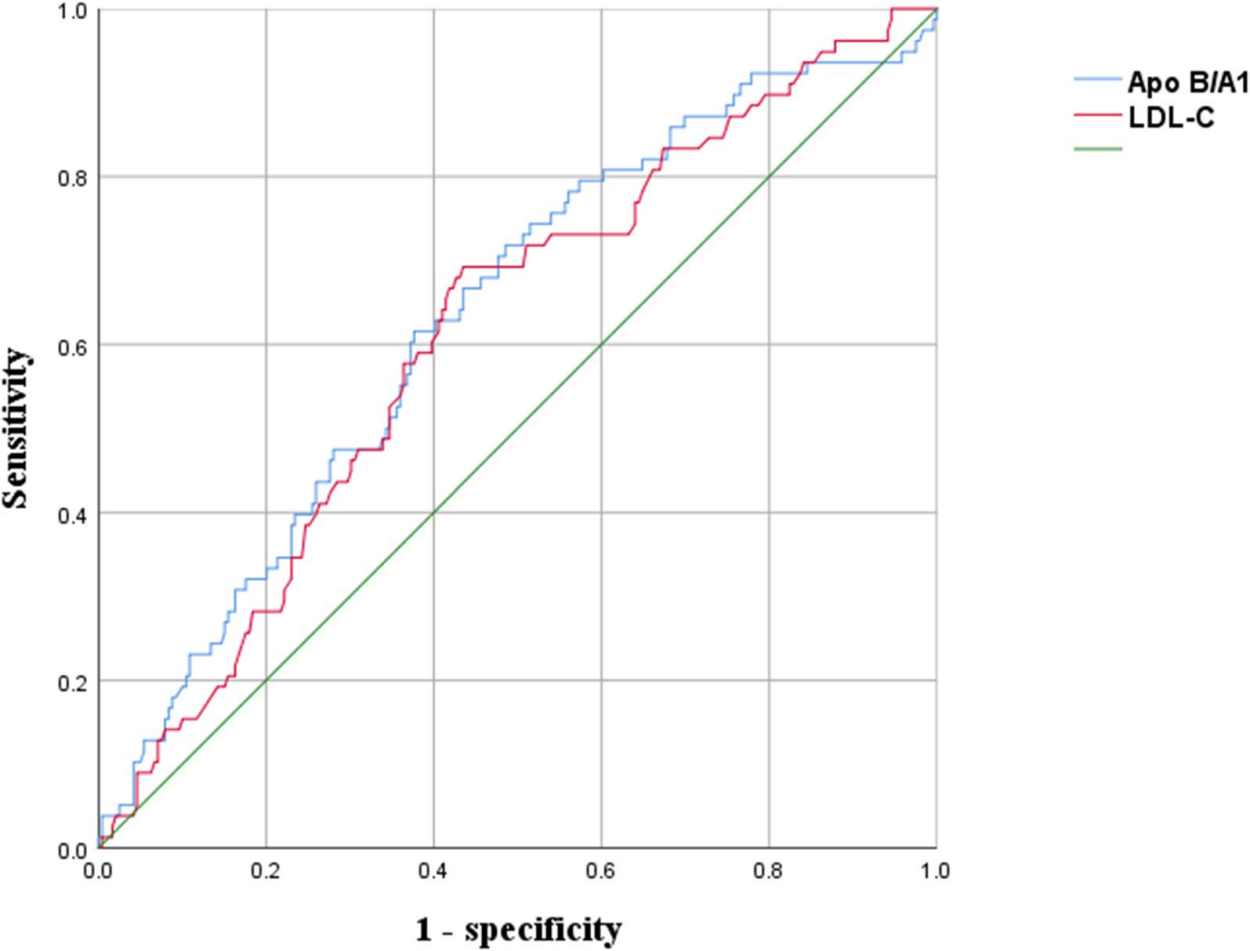
Receiver operating characteristic (ROC) curve for differentiating rupture group from non-rupture group. *AUC* area under the curve, *LDL-C* low-density lipoprotein cholesterol

The predictive value of the Apo B/A1 ratio and LDL-C for vulnerable plaques was further examined by ROC curve analysis. In plaque rupture group, the area under the curve (AUC) were 0.632 (95% CI 0.562–0.702, P < 0.001) for Apo B/A1 and 0.614 (95% CI 0.544–0.683, P = 0.003) for LDL-C (Fig. 4). In plaque erosion group, AUC were 0.624 (95% CI 0.538–0.710, P < 0.001) for Apo B/A1 and 0.605 (95% CI 0.520–0.691, P = 0.027) for LDL-C (Additional file 3: Fig. S1). In thrombus group, AUC were 0.648 (95% CI 0.579–0.716, P < 0.001) for Apo B/A1 and 0.614 (95% CI 0.545–0.682, P = 0.002) for LDL-C (Additional file 4: Fig. S2).

## Discussion

In the present study, the association between the Apo B/A1 ratio and OCT characteristics of coronary vulnerable plaques in patients with ASCVD were investigated. We found that the Apo B/A1 ratio was higher in patients with plaque rupture, erosion, thrombus, and calcification but not calcified nodules, indicating that Apo B/A1 may be associated with coronary vulnerable plaques. The high Apo B/A1 ratio group was associated with high percent of plaque rupture, erosion, and thrombus. Moreover, we revealed that the Apo B/A1 ratio is an independent factor of culprit vulnerable plaques in the method of logistic regression analyses. Apo B/A1 ratio also demonstrates better predictive value of coronary vulnerable plaques compared with LDL-C.

### Apolipoprotein and ASCVD

In recent years, Apos have been found to be strongly associated with the risk of STEMI, and Apo B/A1 ratio is a better risk biomarker of acute myocardial infarction than LDL-C or TC/HDL-C ratio [8, 9]. A low LDL-C/Apo B ratio was found to be associated with neointimal proliferation and neointimal instability after everolimus-eluting stent implantations [10]. A cross-sectional study found that combined evaluation of triglyceride-rich lipoprotein-related markers and the LDL-C/Apo B ratio may be of increasing importance in the risk stratification of ASCVD patients with DM [11].

The Apo B/A1 ratio is a useful tool of risk assessment in patients presenting with NSTEMI including prediction of coronary multi-vessel affection [12]. It is also an independent predictor for complicated lesions and future myocardial infarction in patients with DM and ACS [3]. In the present study, we also found that Apo A1, Apo B, and Apo B/A1 ratio showed a significant difference between ACS and CCS groups, which was consistent with previous study.

### OCT findings and ASCVD

Atherosclerotic plaque components such as TCFA, macrophage infiltration, large necrotic core, and thrombus in patients with ASCVD can be detected with high-resolution imaging modalities including IVUS and OCT [13]. Given its extremely high resolution (< 10 μm) similar to histological biopsy, OCT is usually considered as a technique of in vivo optical biopsy. OCT can distinguish plaque rupture, erosion, and other plaque phenotypes by its optical sensor, which improves disease diagnosis and optimizes therapy. Previous studies have found that plaque rupture, erosion, and calcified nodules are the three main causes of ACS [14, 15]. Plaque rupture is responsible for nearly half of patients with ACS, while plaque erosion and calcified nodule account for 1/3 of patients and 2–7% of acute coronary events, respectively [16, 17]. Other causes of ACS include spontaneous dissection, tight stenosis, and intramural hematoma [18].

Plaque rupture is often associated with large lipid-rich plaque burden and red thrombus, while white thrombus is usually found in plaque erosion [19]. A large study found that approximately 2/3 of plaque erosion cases presented as NSTEMI [20]. However, a meta-analysis revealed that plaque rupture is responsible for 70% of STEMI, 56% of NSTEMI, 39% of unstable angina, and 6% patients of stable angina [21]. In addition, high prevalence of plaque rupture was observed in long duration of DM and high glycosylated hemoglobin (HbA1c) in DM patients with STEMI [22].

In this study, the percentage of plaque rupture was higher than plaque erosion and calcified nodule in patients with ASCVD, while the Apo B/A1 ratio in plaque rupture or erosion groups were both higher in ACS than that of CCS patients (rupture: 36.0 vs. 16.0% and erosion: 22.3 vs. 6.6%). For thrombus, white thrombus was more commonly detected than red thrombus (16.6 vs. 2.2%). The possible reason may be that the percentage of OCT usage in STEMI patients with totally occluded vessel featured with red thrombus was less than that in NSTEMI patients in our study.

To some extent, plaque erosion may overlap with plaque rupture in the setting of TCFA or thick-cap fibroatheroma, which means that plaque erosion with underlying TCFA might harbor small ruptures [23]. Cholesterol crystals, a hallmark of advanced atherosclerotic lesions, were found to be associated with characteristics of vulnerable plaques in ACS culprit lesions [24, 25]. Abundant and homogeneous macrophage accumulation in coronary arterial wall was also found in ACS patients with DM [26]. Another study investigated non-culprit plaque in STEMI patients, and found that STEMI patients with culprit plaque erosion have a limited pancoronary vulnerability than STEMI patients with plaque rupture, which may also explain the better outcomes in patients with erosion than rupture [27]. Patients with plaque erosion had fewer other coronary risk factors (dyslipidemia, hypertension, chronic kidney disease, and DM) than those with plaque rupture [28]. On the basis of the difference of the pathogenesis of plaque rupture and erosion, ACS patients with plaque rupture should be treated with stent implantation, while those with plaque erosion should be treated with antithrombotic and antiplatelet therapy. Our study also revealed that more stents were implanted in ACS patients with plaque rupture than that in CCS group.

### Biomarkers and vulnerable plaques

Although OCT has many advantages, it is ultimately an invasive procedure with low accessibility, high cost, and possible complication risks. Thus, biomarkers that can identify vulnerable plaques in patients with ACS need to be developed. Some serum biomarkers have already been identified to be related to plaque rupture or erosion [29]. High HbA1c and random plasma glucose on admission were found to be positively correlated with vulnerable plaque in STEMI patients [30]. Matrix metalloproteinase-9 (MMP-9), also known as gelatinase B or 92 kDa type IV collagenase, is expressed in atherosclerotic plaques and associated with the vulnerability of plaques including TCFA [31, 32]. MMP-9 can serve as a marker for plaque rupture and predictor of poor clinical outcomes in ACS patients [33]. Low adiponectin level has been shown to be a risk factor for adverse cardiovascular events as adiponectin is involved in the pathogenesis of vulnerability to coronary lesions [34, 35]. Moreover, a significant correlation was observed between low adiponectin levels and plaque rupture, TCFA, and lipid-rich plaque [36]. Serum 1,5-anhydroglucitol also identified high risk for coronary plaque rupture in diabetic patients with ACS [37]. Plasma pentraxin-3 level was associated with plaque vulnerability in patients with ASCVD [38, 39]. Another study indicated that a low ratio of eicosapentaenoic acid to arachidonic acid was associated with higher vulnerability of coronary plaques to rupture [40]. The triglyceride glucose index combined with the morphological characteristics of plaque could be used to predict adverse events in patients with STEMI [41]. The combination of different biomarkers, such as soluble lectin-like oxidized LDL receptor-1, MMP-9, WBC count, and peak creatine kinase-MB, could also be used to identify plaque rupture compared with other individual biomarkers in patients with ACS [18].

It has been proved that a high level of LDL-C is a key risk factor for ASCVD [42]. Many clinical guidelines have shown that statins lower the incidence of ASCVD by reducing the levels of LDL-C, lipid content of plaques, and FCT [43, 44]. Patients with low level of LDL-C who were treated with statins had a higher prevalence of calcification, which means that statins may help to prevent plaque rupture by calcification [45]. However, the addition of proprotein convertase subtilisin/kexin type 9 inhibitor to statin therapy might produce incremental growth in FCT and regression of the lipid-rich plaque after onset of ACS, thus decreasing the incidence of plaque rupture [46].

In terms of lipoprotein and vulnerable plaque phenotype, the reduction of LDL-C and high-sensitivity C-reactive protein was related to changes in total atheroma volume and FCT of residual non-culprit lipid-rich plaques [47]. ACS patients with residual cholesterol risk were found to be associated with atherosclerotic plaque and plaque rupture, indicating that residual cholesterol risk was an independent risk factor of plaque rupture [48]. The levels of lipoprotein (a), an independent risk factor for ASCVD, are associated with an increased atherosclerotic burden, such as higher prevalence of lipidic plaque, wider lipid arc, and higher prevalence of TCFA [49]. Lowering lipoprotein (a) levels by alirocumab contributed independently to cardiovascular event reduction [50]. We found that the Apo B/A1 ratio has a negative correlation with FCT in lipid-rich plaque, indicating the higher Apo B/A1 ratio, the less stable status of TCFA in lipid-rich plaques. Lowering the Apo B/A1 ratio may decrease the incidence of presentation of TCFA and ACS, which need more work to be done in the future. In this study, the ROC curve of Apo B/A1 and LDL-C indicated good sensitivity and specificity for coronary vulnerable plaques. However, the AUC of ROC curve of Apo B/A1 is larger than LDL-C. On the basis of these results, the Apo B/A1 ratio may serve as a better biomarker for vulnerable plaques in patients with ASCVD compared with LDL-C.

### Study limitations

This study has several limitations. First, this study was a retrospective and observational study conducted in one center with a small sample size. Second, we did not classify STEMI, NSTEMI, and UA in the study. Third, most samples were obtained at the time of hospitalization before OCT examination, but a number of blood samples were obtained after that, especially in STEMI and critical patients with high-risk. Fourth, the decision to perform OCT was up to different operators, and potential selection bias may not be excluded. Fifth, calcified nodules were rare in our study. More patients should be enrolled in the future studies.

## Conclusions

We found that the Apo B/A1 ratio was higher in ACS patients than that of CCS patients, and it was associated with coronary culprit plaques, including plaque rupture, erosion, and thrombus. The Apo B/A1 ratio is an independent predictor for coronary vulnerable plaques in patients with ASCVD.

## Supplementary Information

**Additional file 1: Table S1**. Logistic regression analysis of plaque erosion.

**Additional file 2: Table S2**. Logistic regression analysis of thrombus.

**Additional file 3: Fig. S1**. Receiver operating characteristic (ROC) curve for differentiating erosion group from non-erosion group. AUC: area under the curve; LDL-C, low-density lipoprotein cholesterol.

**Additional file 4: Fig. S2**. Receiver operating characteristic (ROC) curve for differentiating thrombus group from non-thrombus group. AUC: area under the curve; LDL-C, low-density lipoprotein cholesterol.

## Data Availability

The datasets used and/or analyzed during the current study are available from the corresponding author on reasonable request.

## Abbreviations

Apo: Apolipoprotein
ASCVD: Atherosclerotic cardiovascular disease
ACS: Acute coronary syndrome
CCS: Chronic coronary syndrome
DM: Diabetes mellitus
OCT: Optical coherence tomography
LDL-C: Low-density lipoprotein cholesterol
HDL-C: High-density lipoprotein cholesterol
PCI: Percutaneous coronary intervention
hs-cTnI: High-sensitivity cardiac troponin I
FCT: Fibrous cap thickness
TCFA: Thin-cap fibroatheroma
STEMI: ST-segment elevation myocardial infarction
NSTEMI: Non-ST-segment elevation myocardial infarction
UA: Unstable angina
WBC: White blood cells
TC: Total cholesterol
TG: Total triglycerides
ROC: Receiver operator characteristic
AUC: Area under the curve
MMP-9: Matrix metalloproteinase-9
