# Supplementary material for: Association between apolipoprotein B/A1 ratio and coronary plaque vulnerability in patients with atherosclerotic cardiovascular disease: an intravascular optical coherence tomography study
Source: Cardiovasc Diabetol. 2021 Sep 15;20:188. doi: 10.1186/s12933-021-01381-9 (PMC8442358; doi:10.1186/s12933-021-01381-9)
Supplement: Supplementary file 1 — Additional file 1: Table S1. Logistic regression analysis of plaque erosion. [file 12933_2021_1381_MOESM1_ESM.docx]

**Table S1. Logistic regression analysis of plaque erosion**

| **Variables** | **OR** | **95% CI** | **P value** |
| --- | --- | --- | --- |
| Apo B/A1 | 3.659 | 1,140-11.743 | 0.029 |
| Apo B | 2.982 | 0.865-10.285 | 0.084 |
| Apo A1 | 0.419 | 0.107-1.650 | 0.214 |
| TG | 1.252 | 1.040-1.509 | 0.018 |
| TC | 1.354 | 0.998-1.839 | 0.052 |
| HDL-C | 0.415 | 0.108-1.594 | 0.200 |
| LDL-C | 1.404 | 0.974-2.026 | 0.069 |
| VLDL-C | 1.744 | 1.017-2.992 | 0.043 |
| Lipoprotein (a) | 0.998 | 0.987-1.010 | 0.774 |
| Model 1 | 3.874 | 1.185-12.665 | 0.025 |
| Model 2 | 2.792 | 0.818-9.530 | 0.101 |
| Model 3 | 2.840 | 0.837-9.642 | 0.094 |

Model 1: Apo B/A1, AF, HBP and DM.

Model 2: Apo B/A1, AF, HBP, DM, age and sex.

Model 3: Apo B/A1, AF, HBP, DM, age, sex, alcohol drinking and smoking.

Apo, apolipoprotein; TG, total triglycerides; TC, total cholesterol; HDL-C, high-density lipoprotein cholesterol; LDL-C, low-density lipoprotein cholesterol; VLDL-C, very low-density lipoprotein cholesterol; OR. odds ratio; CI, confidence interval.
