# Supplementary material for: Association between apolipoprotein B/A1 ratio and coronary plaque vulnerability in patients with atherosclerotic cardiovascular disease: an intravascular optical coherence tomography study
Source: Cardiovasc Diabetol. 2021 Sep 15;20:188. doi: 10.1186/s12933-021-01381-9 (PMC8442358; doi:10.1186/s12933-021-01381-9)
Supplement: Supplementary file 2 — Additional file 2: Table S2. Logistic regression analysis of thrombus. [file 12933_2021_1381_MOESM2_ESM.docx]

**Table S2. Logistic regression analysis of thrombus**

| **Variables** | **OR** | **95% CI** | **P value** |
| --- | --- | --- | --- |
| Apo B/A1 | 7.326 | 2.692-19.940 | <0.001 |
| Apo B | 4.900 | 1.813-13.243 | 0.002 |
| Apo A1 | 0.324 | 0.113-0.928 | 0.036 |
| TG | 1.174 | 0.985-1.398 | 0.073 |
| TC | 1.405 | 1.097-1.800 | 0.007 |
| HDL-C | 0.370 | 0.134-1.027 | 0.056 |
| LDL-C | 1.545 | 1.153-2.070 | 0.004 |
| VLDL-C | 1.555 | 0.937-2.581 | 0.087 |
| Lipoprotein (a) | 1.002 | 0.993-1.010 | 0.679 |
| Model 1 | 8.777 | 3.121-24.679 | <0.001 |
| Model 2 | 6.147 | 2.145-17.613 | 0.001 |
| Model 3 | 6.791 | 2.304-20.013 | 0.001 |

Model 1: Apo B/A1, AF, HBP and DM.

Model 2: Apo B/A1, AF, HBP, DM, age and sex.

Model 3: Apo B/A1, AF, HBP, DM, age, sex, alcohol drinking and smoking.

Apo, apolipoprotein; TG, total triglycerides; TC, total cholesterol; HDL-C, high-density lipoprotein cholesterol; LDL-C, low-density lipoprotein cholesterol; VLDL-C, very low-density lipoprotein cholesterol; OR. odds ratio; CI, confidence interval.
