# Supplementary figures and images for: Association between apolipoprotein B/A1 ratio and coronary plaque vulnerability in patients with atherosclerotic cardiovascular disease: an intravascular optical coherence tomography study
Source: Cardiovasc Diabetol. 2021 Sep 15;20:188. doi: 10.1186/s12933-021-01381-9 (PMC8442358; doi:10.1186/s12933-021-01381-9)

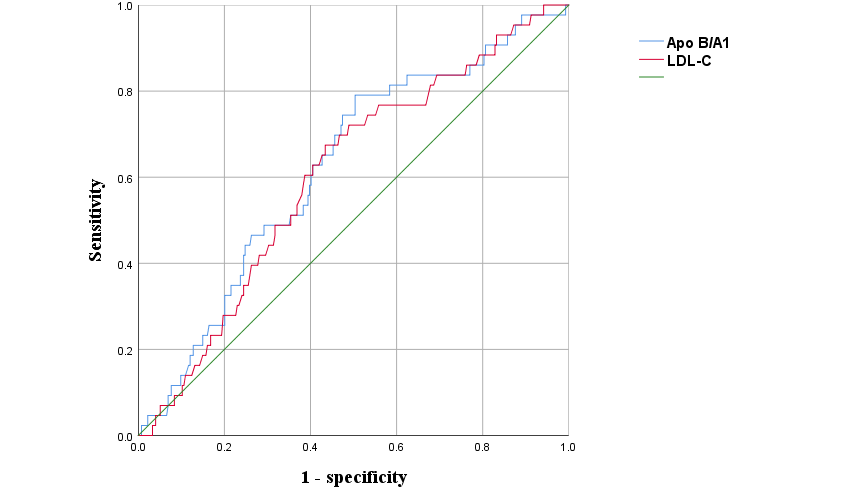

Supplement: Supplementary file 3 — Additional file 3: Fig. S1. Receiver operating characteristic (ROC) curve for differentiating erosion group from non-erosion group. AUC: area under the curve; LDL-C, low-density lipoprotein cholesterol. [file 12933_2021_1381_MOESM3_ESM.tif]

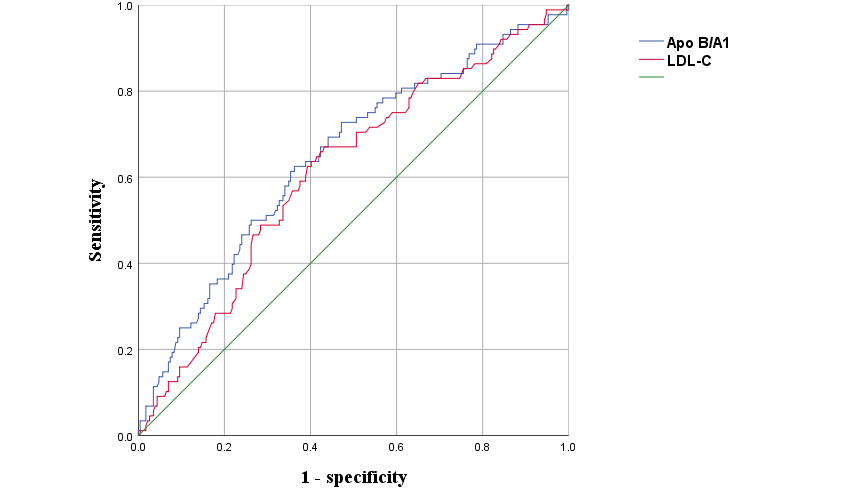

Supplement: Supplementary file 4 — Additional file 4: Fig. S2. Receiver operating characteristic (ROC) curve for differentiating thrombus group from non-thrombus group. AUC: area under the curve; LDL-C, low-density lipoprotein cholesterol. [file 12933_2021_1381_MOESM4_ESM.tif]
